# ANGPTL4 regulate glutamine metabolism and fatty acid oxidation in nonsmall cell lung cancer cells

**DOI:** 10.1111/jcmm.16879

**Published:** 2022-03-13

**Authors:** Song Xiao, Wang Nai‐dong, Yan Jin‐Xiang, Tian Long, Lu Xiu‐Rong, Gao Hong, Yan Jie‐Cheng, Zhang Fei

**Affiliations:** ^1^ Radiotherapy Department the First Affiliated Hospital of Hebei North University Zhangjiakou China; ^2^ Department of Pharmacy Ji Nan Hospital Jinan China; ^3^ Neurosurgery Department Ningyang NO.1 People's Hospital Ningyang China; ^4^ Radiotherapy Department Beijing Hospital Beijing China

**Keywords:** aerobic glycolysis, ANGPTL4, fatty acid oxidation, glutamine, NSCLC

## Abstract

Angiopoietin‐like protein (ANGPTL) 4 is a key factor in the regulation of lipid and glucose metabolism in metabolic diseases. ANGPTL4 is highly expressed in various cancers, but the regulation of energy metabolism in tumours remains to be determined. This study explored the role of ANGPTL4 in aerobic glycolysis, glutamine consumption and fatty acid oxidation in nonsmall cell lung cancer (NSCLC) cells. Two NSCLC cell lines (A549 and H1299) were used to investigate the role of ANGPTL4 in energy metabolism by tracer techniques and with Seahorse XF technology in ANGPTLs4 knockdown cells. RNA microarrays and specific inhibitors were used to identify targets in ANGPTLs4‐overexpressing cells. The results showed that knockdown of ANGPTLs4 could inhibit energy metabolism and proliferation in NSCLC. ANGPTLs4 had no significant effect on glycolysis but affected glutamine consumption and fatty acid oxidation. Knockdown of ANGPTLs4 also significantly inhibited tumour metastasis and energy metabolism in mice and had a weak effect on glycolysis. RNA microarray analysis showed that ANGPTLs4 significantly affected glutaminase (GLS) and carnitine palmitoyl transferase 1 (CPT1). ANGPTLs4‐overexpressing cells were exposed to a glutamine deprivation environment, and cell proliferation and energy metabolism were significantly decreased but still differed from normal NSCLC cells. Treatment of ANGPTLs4‐overexpressing cells with GLS and CPT1 inhibitors simultaneously prevented the regulatory effects on cell proliferation and energy metabolism. ANGPTLs4 could promote glutamine consumption and fatty acid oxidation but not glycolysis or accelerate energy metabolism in NSCLC.

## 1 INTRODUCTION

Lung cancer is the most common cause of cancer death in the world, with an estimated 1.6 million deaths per year. From the clinical treatment and biological characteristics, lung cancer is mainly divided into small cell lung cancer and nonsmall cell lung cancer. Approximately 85% of patients are collectively referred to as nonsmall cell lung cancer (NSCLC). Abnormal energy metabolism is a characteristic of tumours.^1^, ^2^ The classical example of a reprogrammed metabolic pathway in cancer is the Warburg effect.^3^ De novo lipid synthesis and glutaminolysis are also important components in cancer cell metabolic reprogramming.^4^, ^5^ Tumour cells promote energy metabolism to meet the requirements of cell biosynthesis and cell function.

Angiopoietin‐like protein 4 (ANGPTL4) is a multifunctional cytokine that is involved in both angiogenesis and metastasis.^6^ ANGPTL4 exhibits structural similarity to multifunctional angiopoietins, which are involved in glucose homeostasis, lipid metabolism, angiogenesis, inflammation, and tumour progression and metastasis.^7^, ^8^, ^9^ ANGPTL4 plays an important role in the tumour microenvironment, especially in hypoxia induction. Therefore, in recent years, research on ANGPTL4 in tumour function has found that activation of ANGPTL4 in lung cancer cells by hypoxia inducible factor‐α (HIF‐α) can promote tumour cell proliferation.^10^ ANGPTL4 deficiency by genetic knockdown or treatment with a neutralizing antibody led to a significant reduction in obesity‐induced angiogenesis and tumour growth.^11^ However, how ANGPTL4 participates in tumour cell energy metabolism, especially its mechanism in NSCLC, remains to be elucidated.

## 2 MATERIALS AND METHODS

### 2.1 Cells culture

Two NSCLC cell lines (A549, H1299) was purchased from the cell bank of the Chinese academy of sciences (Shanghai, China). NSCLC cells were maintained in Dulbecco's Modified Eagle Medium (DMEM) (Gibco, Grand Island, NY, USA) supplemented with 10% foetal bovine serum (FBS; Gibco), penicillin (100 U/ml) and streptomycin (100 µg/ml) (Beyotime Biotech, Haimen, China) in humidified air at 37℃ with 5% CO_2_.

### 2.2 Knockdown/overexpression of ANGPTL4 in NSCLC

To knockdown ANGPTL4 expression, siRNA (Thermo Fisher Scientific) was transfected using RNAiMax (Invitrogen, Carlsbad, CA) according to the manufacturer's recommendations. The sequence of the siRNAs was referred to existing reports.^12^ Control cells were transfected with scrambled siRNA (Thermo Fisher Scientific). ANGPTL4 forward 5’‐AGACACAACTCAAGGCTCAG‐3′ and ANGPTL4 reverse 5′‐CTCATGGTCTAGGTGCTTGTG‐3′; Overexpression of ANGPTL4 cells were transfected in A549 cells (Santa Cruz, USA) to increase the level of ANGPTL4(S‐5’‐TCTCTCACCGGGTATGAGCGGTGCTCCGACGGCC‐3’, AS‐5’‐GTGTCTTAATTAACTAGGAGGCTGCCTCTGCTGC‐3’). Full‐length cDNA encoding human ANGPTL4 were cloned into the vector plasmid (Shanghai Genechem Co., Ltd).

### 2.3 Glutamine deprivation cell model

Glutamine deprivation cell model was established follow the method below, remove normal DMEM, and wash normal NSCLC cells or transfect cells with phosphate buffer saline (PBS). Adding DMEM (ThermoFisher Science, A14431) which without glutamine. In addition, 10% foetal bovine serum (Gemini Bio Products, Sacramento, California, USA) was added to the medium. The cells were cultured in this medium for 24 hours and continued to be used.

### 2.4 Cell proliferation assay with cell counting Kit‐8 (CCK‐8)

96‐well plate was used to implant cells. The initial cell concentration was 5000 cells/pore, and the cell proliferation activity was measured by CCK‐8 (Beyotime Biotech, Haimen, China). CCK‐8 reagent was added to the pore after 24, 48, 72 and 96 hours of cell growth. The cells were cultured in cell culture box for 1.5 hours. Absorption was measured at 450 nm to investigate the cell proliferation activity.

### 2.5 Animal and tumour model

Six‐ to eight‐week male nude mice were obtained from the Hebei Medical University experimental animal centre. The mice were housed in a specific pathogen‐free facility with free access to normal chow and water. Individual BALB/c nude mice were inoculated subcutaneously with A549 cells (1 × 10^6^ cells in 0.1 ml of PBS) on the right leg. When a tumour volume reached~50 mm^3^, individual mice were randomized and treated intravenously with 0.1 ml of 5% glucose solution through the caudal vein as a vehicle, 10 μg liposomeencapsulated pshRNA‐Con or pshRNA‐ ANGPTL4 A549 cells. Their body weights and tumour growth were measured every day, and the tumour volumes (V) were calculated using the formula: V = a × b^2/2^, where a and b are the largest and smallest tumour diameter, respectively. The mice were sacrificed 24 hours after the final treatment. Each experiment was performed twice.

### 2.6 Glutamic acid, acetyl coa, Adenosine triphosphate (ATP) and lactate production

Normal or transfected cells were inoculated into 6‐well plates. After washing with PBS, the cells were digested with 0.25% trypsin and the protein content was quantified by BCA kit. The Glutamic acid (Jiancheng Nanjing, A073‐1–1), acetyl coa (Jiancheng Nanjing, A012‐1), ATP (Biovision, K354‐100) and lactic acid (Biovision, K462) content in cells and tumour model in mice were measured by spectrophotometer and commercial kit.

### 2.7 Isotope tracing metabolomics of glutamine and fatty acid oxidation (FAO)

The isotope tracing metabolomics was used. We used DMEM medium that was glucose‐ and glutamine‐free (Gibco, Thermo Fisher Scientific, USA), which supplemented with 5mM 13C_5_‐glutamine (Sigma) and ^13^C16‐palmitate. Following 16 hours of incubation, cells were harvested and the metabolites extracted with ice‐cold methanol and α‐KG (M+5), malate (M+4), aspartate (M+4) [13C_16_]‐acetyl‐CoA and [13C_16_]‐acetylcarnitine were measured by LC/MS as described.^13^

### 2.8 Gene detection and Western blotting

Total RNA was extracted from cells using TRIzol reagent (Thermo Fisher Scientific). The purity of RNA was determined by NanoDrop ND‐1000 spectrophotometer (Thermo Scientific) qPCR was conducted using the appropriate primers and a Bio‐Rad CFX96 system with SYBR green to determine the mRNA expression levels of genes in cells. GAPDH was used as endogenous controls and further analysed by the 2^∆DDCT^ method. All the primers used for qPCR are listed in the supplement data.

Total proteins were isolated from A549 and H1299 cells and dissolved in Ripa buffer which containing protease inhibitor (sigma, USA). The total protein concentration was determined by BCA analysis kit (Rockford Pierce, Illinois, USA). Total protein samples (30 mg) were analysed by 10% SDS‐PAGE gel and transferred to PVDF membrane. After the blocking procedure, the cell membrane was incubated overnight with primary antibody and secondary antibodies. Then, visualized in Imager (Bio‐Rad) using ECL system (Thermo Fisher Scientific, 34095). The following antibodies were used: ANGPTL4 (1:5000, abcam, MA, USA), GLS (1:1000, abcam, MA, USA), CPT1 (1:1000, abcam, MA, USA) and GAPDH (1:5000, abcam, MA, USA).

### 2.9 Statistical analyses

All experimental results were analysed by SPSS 17.0 software and expressed as mean ±SEM. Standard ANOVA procedures followed by multiple pairwise comparison adjusted with Bonferroni corrections were performed for cell viability assays. Unpaired Student's t tests were used to analyse all the other results. Significance was considered at *P* < 0.05.

## 3 RESULTS

### 3.1 ANGPTL4 promotes OXPHOS but not aerobic glycolysis

**FIGURE 1:**
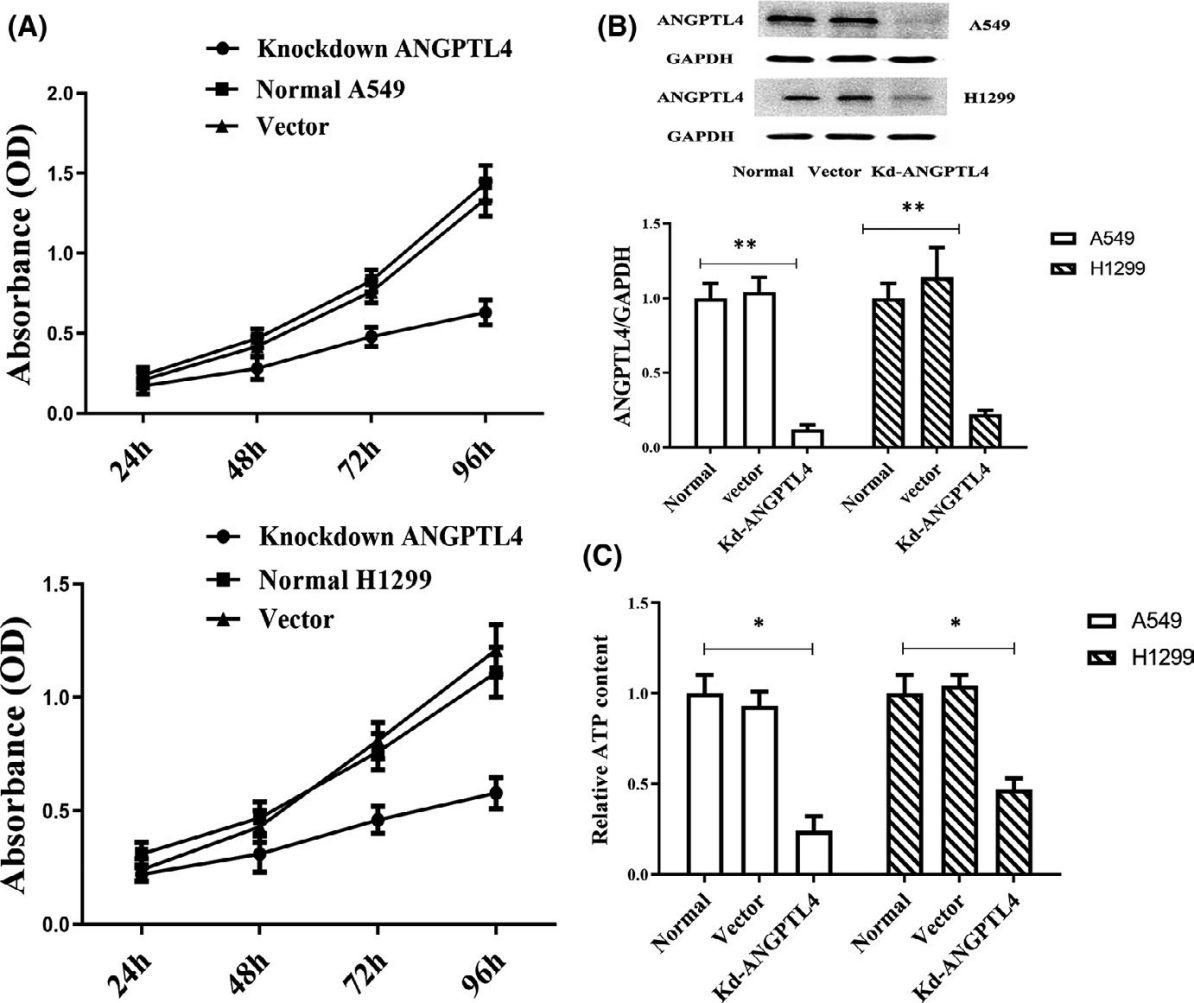
ANGPTL4 knockdown reduces the proliferation and ATP content of A549 and H1299 cells. (A) The viability of normal NSCLC and ANGPTL4 knockdown cells was analysed by CCK in A549 cells and H1299 cell. (B) The expression of ANGPTL4 in normal NSCLC, ANGPTL4 vector and ANGPTL4 knockdown cells were determined by Western blot analysis of protein level. **P < 0.01. (C) The ATP content in normal NSCLC (ANGPTL4 vector transfection cell) and ANGPTL4 knockdown cells was analysed. *P < 0.05

To investigate the effect of ANGPTL4 on NSCLC cells, ANGPTL4‐knockdown NSCLC cells were established in A549 and H1299 cells. The results showed that cell proliferation decreased significantly after ANGPTL4 knockdown compared to that in nontreated A549 or H1299 cells (Figure 1A,B). At the same time, ATP was also examined, and the ATP content decreased significantly in ANGPTL4 knockdown cells (Figure 1C).

**FIGURE 2:**
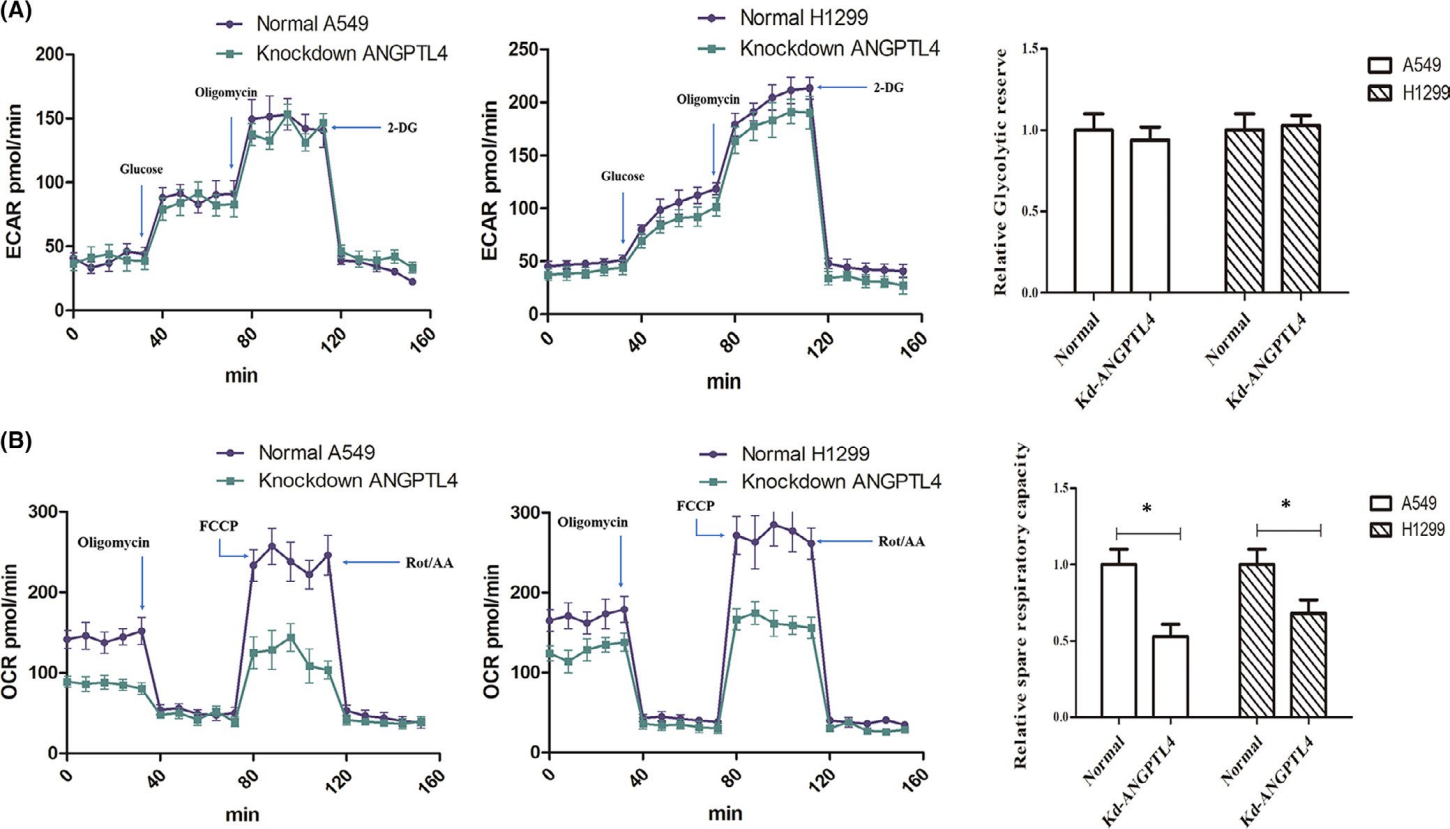
ANGPTL4 perform OXPHOS but not aerobic glycolysis. The OXPHOS and aerobic glycolysis in normal NSCLC (ANGPTL4 vector transfection cell) and ANGPTL4 knockdown cells were determined (A) Representative trace (left) and mean data (centre) of glycolytic ECAR when assayed in minimal medium and treated with glucose in Seahorse. Glycolytic reserve (right) is the difference between glycolytic capacity and glycolysis rate and was measured as the difference between resulting ECAR values after in‐Seahorse exposure to glucose and maximal ECAR values obtained after oligomycin treatment. (B) Representative trace (left) and mean data (centre) of oxygen consumption rate (OCR) from cells as in a. Values are averages of 5–8 basal OCR measurements minus averages of 3–5 OCR readings after rot/AA treatment of individual wells from indicated cell types. Spare respiratory capacity is the difference between basal OCR values and maximal OCR values obtained after FCCP uncoupling (right). *p < 0.05, **p < 0.01, 2DG, 2‐deoxy‐d‐glucose; FCCP, carbonyl cyanide‐4 (trifluoromethoxy) phenylhydrazone; rot/AA, rotenone and antimycin A

Knockdown of ANGPTL4 significantly reduced ATP levels in cells. Therefore, the effect of ANGPTL4 on glycolysis or oxidative phosphorylation (OXPHOX) in NSCLC energy metabolism was investigated. To understand the real‐time state of glycolysis and the mitochondrial oxygen consumption rate (OCR), Seahorse XF technology was utilized, and the OCR and extracellular acidification rate (ECAR) ratios in ANGPTL4 knockdown cells were measured. Following the addition of glucose, the glycolytic rate was not significantly different between ANGPTL4 knockdown cells and normal NSCLC cells. Moreover, glucose consumption and lactate levels were not significantly changed in ANGPTL4 knockdown cell lines (Figure 2A). In contrast, the results showed that the oxygen consumption rate (OCR) in ANGPTL4 knockdown cells was significantly decreased compared to that in normal NSCLC cells (Figure 2B). The results show that the expression of ANGPTL4 could promote energy synthesis and OXPHOS but not aerobic glycolysis in NSCLC cells.

### 3.2 ANGPTL4 promotes glutamine metabolism and fatty acid oxidation

**FIGURE 3:**
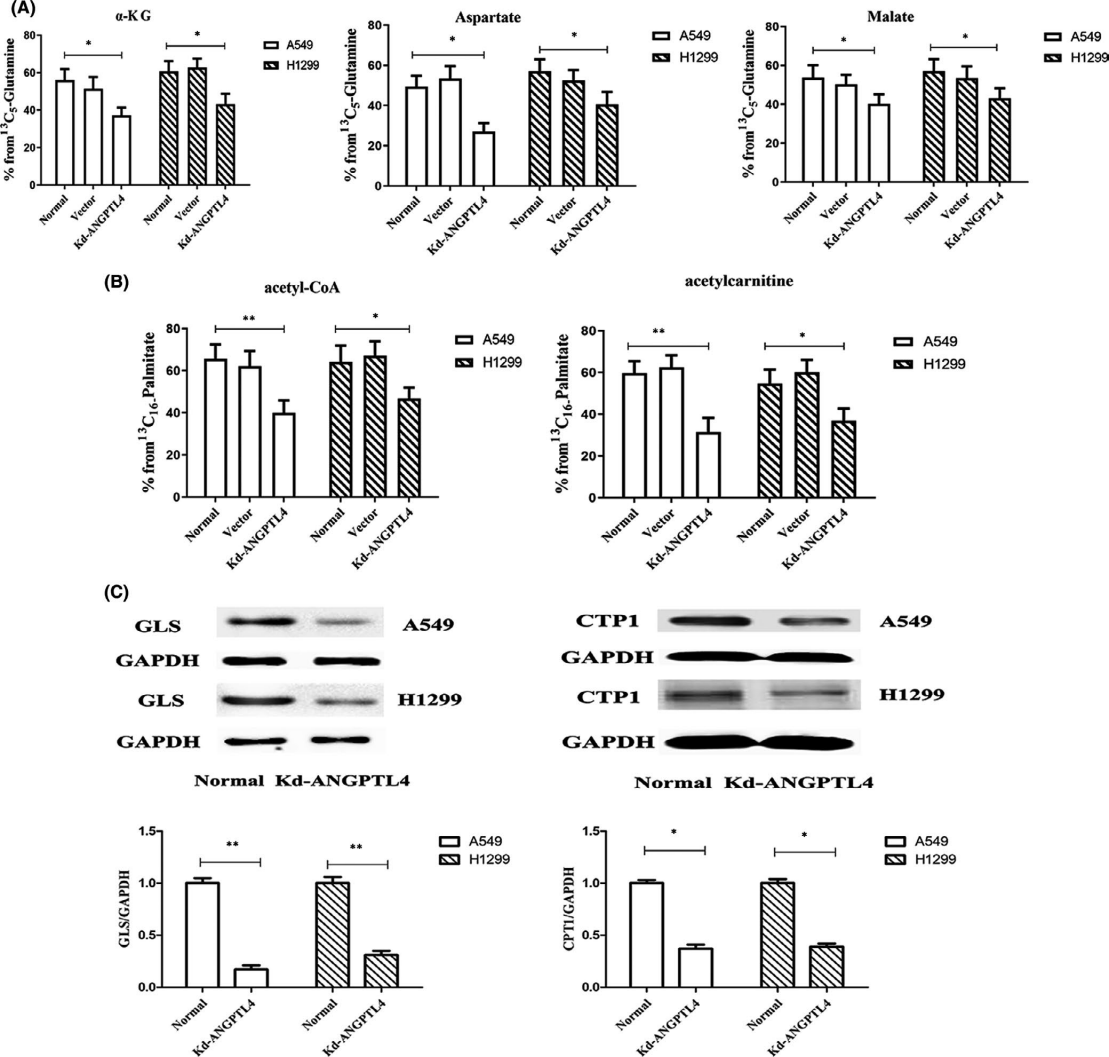
ANGPTL4 promotes glutamine metabolism and fatty acid oxidation. (A) (A) Glutamine consumption determined in NSCLC cells, isotope abundance of α‐KG (M+5), malate (M+4), aspartate (M+4) in normal NSCLC, ANGPTL4 vector and ANGPTL4 knockdown cells were traced by ^13^C_5_‐glutamine. *P < 0.05. (B) Fatty acid oxidation determined in NSCLC cells, the content of [^13^C_16_]‐acetyl‐CoA and [^13^C_16_]‐acetylcarnitine isotope in normal NSCLC, ANGPTL4 vector and ANGPTL4 knockdown cells were traced by ^13^C_16_‐palmitate. *P < 0.05. (C) The expression of GLS and CPT1 in normal NSCLC(ANGPTL4 vector transfection cell) and ANGPTL4 knockdown cells were determined by Western blot analysis of protein level. **P < 0.01. *P < 0.05

The Seahorse XF mito fuel flex test showed that ANGPTL4 promotes OXPHOS but not aerobic glycolysis. Glutamine or fatty acids are the main raw material in OXPHOS. To better understand ANGPTL4‐specific metabolic mechanisms, we analysed the metabolites in vitro by ^13^C labelling in ANGPTL4 knockdown and control cells. ANGPTL4 knockdown and control cells were incubated for 16 h in the presence of 5 mM 13C_5_‐glutamine (Sigma) or 13C_16_‐palmitate. ANGPTL4 knockdown could significantly reduce the related substances that enter the mitochondrial TCA cycle through glutamine metabolism. The related products of glutamine metabolism, including isotope‐encoded α‐KG (M+5), aspartate (M+4) and malate (M+4), were significantly decreased in ANGPTL4 knockdown cells (Figure 3A). Fatty acid metabolism was analysed by detecting acetyl‐CoA from [13C_16_]‐palmitate. After knocking out ANGPTL4, the contents of [13C_16_]‐acetyl‐CoA and [13C_16_]‐acetylcarnitine were also significantly decreased (Figure 3B).

The results of RNA microarray analysis showed that knockdown of ANGPTL4 had certain effects on fatty acid oxidation, glutamine metabolism and glycolysis pathway gene expression (Data S1, Table S1). CPT1 and GLS gene expression was significantly decreased (Data S1, Figure S1). The protein quantification results were consistent with those of the gene chip (Figure 3C).

### 3.3 Knockdown of ANGPTL4 inhibits the growth of implanted tumours in mice

**FIGURE 4:**
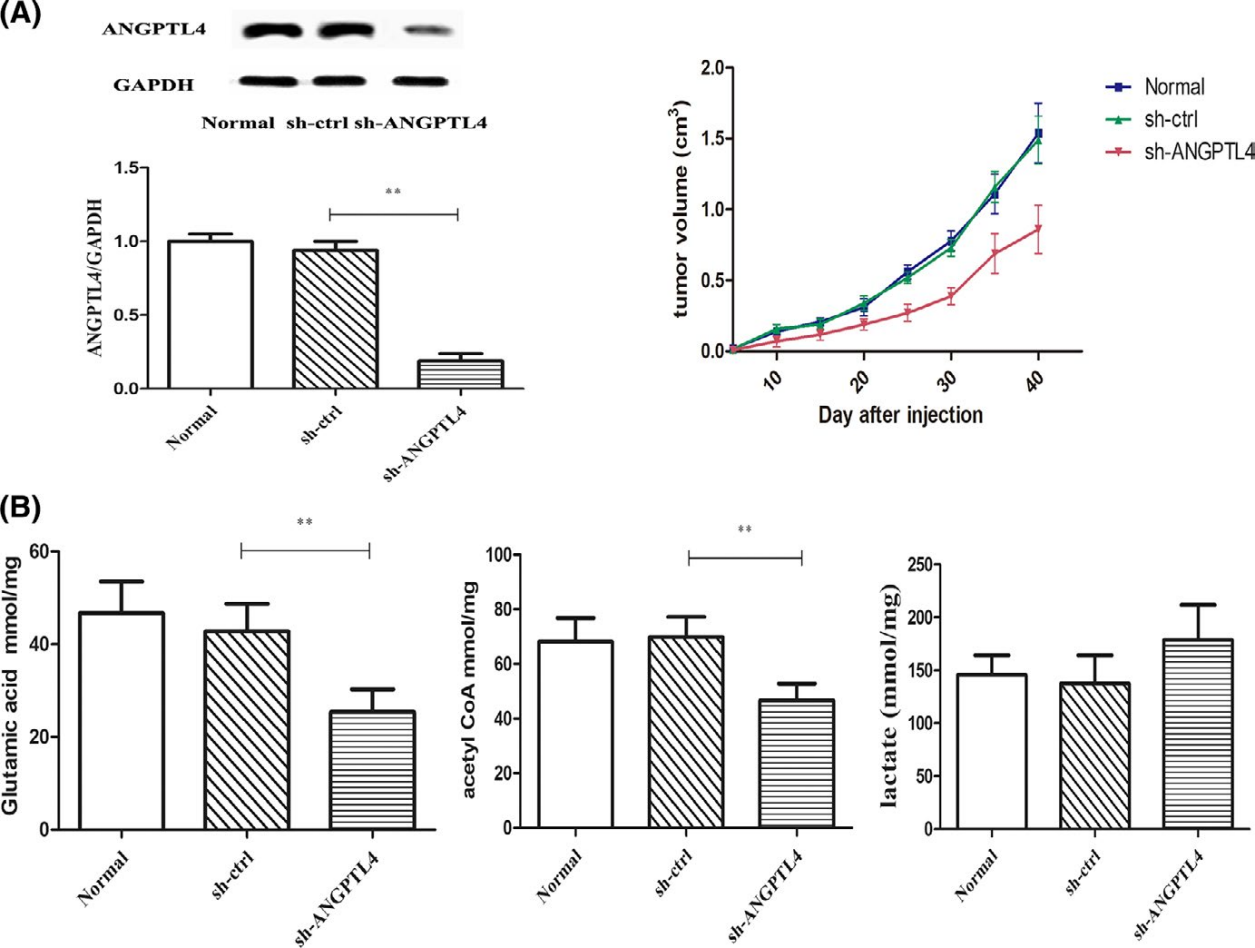
ANGPTL4 knockdown inhibited the tumour growth, glutamine metabolism and fatty acid oxidation in subcutaneous xenograft model. (A). ANGPTL4 in tumour and the tumour volume of subcutaneous xenograft model for NSCLC cells. (B) Glutamic acid, acetyl coa and lactate content were determined in subcutaneous xenograft model. **P < 0.01

To determine the effect of ANGPTL4 silencing on NSCLC and energy metabolism in vivo, nude mice were subcutaneously inoculated with 1 × 10^6^ A549 cells, and when the implanted tumours grew to ^50 mm3^ in one dimension, the mice were randomized and treated intravenously with vehicle as a control, pshRNA‐control or pshRNA‐ANGPTL4. The growth of implanted tumours in individual mice was monitored longitudinally, and the growth of implanted tumours in different groups of mice was indistinguishable at individual time points (Figure 4A). The control plasmid did not affect the growth of implanted tumours in mice, but treatment with pshRNA‐ANGPTL4 significantly inhibited the growth of implanted tumours. The contents of glutathione and acetyl‐CoA in the tumours of nude mice were detected but had little effect on the lactate content. The results showed that the content of glutathione and acetyl‐CoA in the tumour decreased significantly after administration of pshRNA‐ANGPTL4 (Figure 4B).

### 3.4 ANGPTL4 upregulated glutaminase and acetyl‐CoA synthesis in NSCLC

**FIGURE 5:**
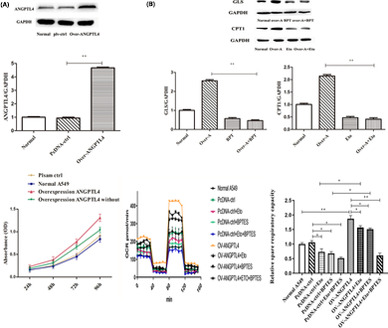
ANGPTL4 overexpression promoted NSCLC proliferation and OXPHOS of A549 cells, but inhibited by CPT1 and GLS inhibitors. (A) ANGPTL4 overexpression promoted NSCLC proliferation. (A) The viability of ANGPTL4 overexpression cells in normal environmental and without glutamine was analysed by CCK in A549 cells. (B) Representative trace and mean data (centre) of oxygen consumption rate (OCR) from ANGPTL4 overexpression cells with CPT1 inhibitor (Etomoxir sodium salt, ETO, 15 μM) or GLS inhibitor (BPTES, 20 μM) were determined. Results are representative of (for sample data) or represent the mean of 3–6 independent experiments. *P < 0.05, **P < 0.01, 2DG, 2‐deoxy‐d‐glucose; FCCP, carbonyl cyanide‐4 (trifluoromethoxy) phenylhydrazone; rot/AA, rotenone and antimycin A

**FIGURE 6:**
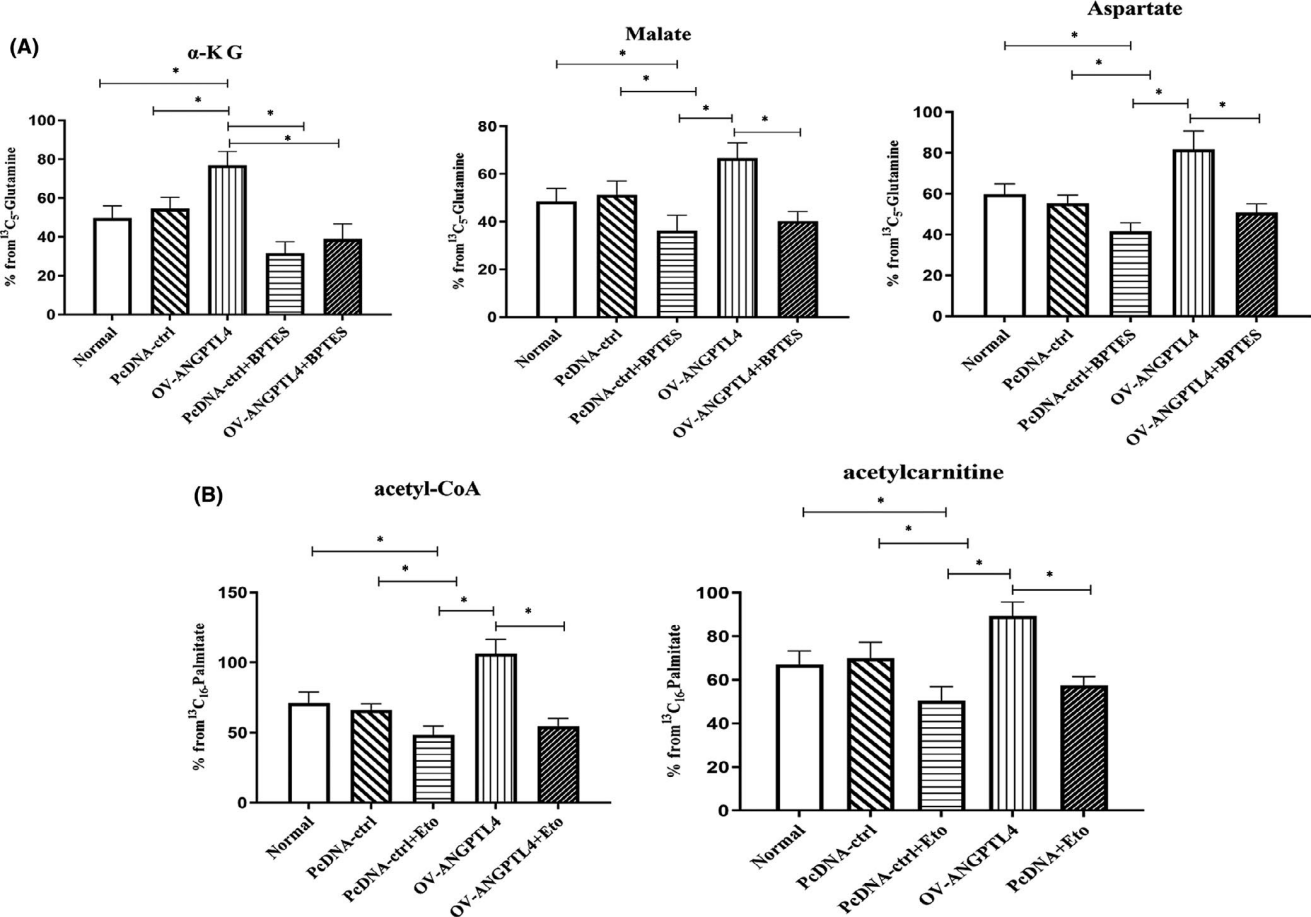
ANGPTL4 overexpression promotes glutamine metabolism and fatty acid oxidation, but inhibited by CPT1 (ETO, 15 μM) and (BPTES, 20 μM). (A) Glutamine consumption determined in NSCLC cells, isotope abundance of α‐KG (M+5), malate (M+4) and aspartate (M+4) in NSCLC cells were traced by ^13^C_5_‐glutamine. *P < 0.05. (B) Fatty acid oxidation determined in NSCLC cells, the content of [^13^C_16_]‐acetyl‐CoA and [^13^C_16_]‐acetylcarnitine isotope were traced by ^13^C_16_‐palmitate. *P < 0.05. NSCLC cell which pretreatment with GLS inhibitor BPTES (20 μM,Selleck) or CPT1 inhibitor ETO (15 μM,Selleck)for 24h and ANGPTL4 overexpression NSCLC cell combined with BPTES or ETO. *P < 0.05

To examine whether ANGPTL4 mediated cell growth through glutamine and fatty acid oxidation dependently, ANGPTL4‐overexpressing cells were cultured in medium with or without glutamine. The results showed that the proliferation of ANGPTL4‐overexpressing A549 cells was decreased in glutamine‐free medium compared to glutamine medium. However, there were still significant differences in the glutamine‐free medium group of ANGPTL4‐overexpressing A549 cells compared with that of normal A549 cells (Figure 5A). The effect of ANGPTL4 on OXPHOS was determined using a CPT1 inhibitor (Eto) or GLS inhibitor (BPTES). Overexpression of ANGPTL4 significantly promoted OXPHOS in NSCLC cells, but this phenomenon decreased significantly when CPT1 or GLS inhibitor was given and disappeared when both the GLS and CTP1 inhibitors were given. There was no significant difference between the two inhibitors (Figure 5B). The effect of ANGPTL4 on glutamine metabolism and fatty acid oxidation of cells was also further investigated by administering CPT1 inhibitor (Eto) and GLS inhibitor (BPTES), respectively. The results showed that when a GLS inhibitor (BPTES) was given, the regulatory effect of ANGPTL4 on glutamine metabolites disappeared, and the related products of glutamine metabolism, including isotope‐encoded a‐KG (M+5), aspartate (M+4) and malate (M+4), were significantly decreased. [13C_16_]‐acetyl‐CoA and [13C_16_]‐acetylcarnitine decreased significantly when a CPT1 inhibitor (Eto) was given, and the regulatory effect on fatty acid metabolites disappeared (Figure 6).

## 4 DISCUSSION

The reorganization of energy metabolism is the power source of tumour cell proliferation. Metabolic reprogramming takes place in sugar metabolism, amino acid metabolism, lipid metabolism and other aspects.^14^ Glycolysis is the main feature of energy metabolism in tumour cells. Pyruvate produced by glycolysis is not coupled with the TCA cycle in the mitochondria but is converted into lactic acid to produce ATP.^12^, ^13^, ^15^ In addition to glycolysis, the mitochondrial TCA cycle is another source of energy for tumour cells. Glutamine is an important raw material for the TCA cycle in tumour cells.^16^ After glutamine is converted into glutamic acid, it is catalysed to α‐ketoglutarate (α‐KG), which enters the tricarboxylic acid cycle to provide a carbon source. In addition, lipid metabolism is also an important energy source for tumour cells. The fatty acid and cholesterol synthesis pathway in tumours is active and includes acetyl‐CoA carboxylase (ACC) and fatty acid synthetase (FASN).^17^, ^18^ High expression of genes related to lipid uptake and transport, lipid synthesis, intracellular distribution and fatty acid oxidation was found to be involved in highly metastatic tumour cells.^19^

The angiopoietin‐like 4 (ANGPTL4) protein belongs to a superfamily of secreted proteins structurally related to factors modulating angiogenesis known as angiopoietins. This protein family includes eight members encoded by eight genes (ANGPTL1‐8) identified in humans and mice.^20^ ANGPTL4 is highly expressed in breast cancer, colorectal cancer, prostate cancer, liver cancer, kidney cancer and other tumours and participates in the regulation of tumour growth, redox reactions, angiogenesis, metastasis and other biological functions. Although studies have shown that the high expression of ANGPTL4 in breast cancer is beneficial to inhibit the proliferation of triple‐negative breast cancer,^21^ more studies have shown that ANGPTL4 has a significant effect on lung cancer cell proliferation.^22^ Recent research indicated that ANGPTL4 also participates in lipid metabolism and regulates cell energy metabolism.^23^ ANGPTL4 deficiency in macrophages results in ER stress due to the cell‐intrinsic reprogramming of fatty acid metabolism.^22^ ANGPTL4 has surfaced as a principal regulator of plasma lipid metabolism by functioning as a potent inhibitor of lipoprotein lipase in cardiovascular disease.^24^

Through gene knockdown technology, we found that knockdown of ANGPTL4 in different NSCLC cells significantly decreased cell proliferation and energy metabolism, which indicated that ANGPTL4 could affect the proliferation ability of NSCLC cells by participating in energy metabolism. With Seahorse XF technology, the differences in oxidative phosphorylation and glucose‐mediated cell acidification metabolism in NSCLC cells were analysed after ANGPTL4 knockdown. The experimental results showed that compared with the characteristic glycolysis of tumour cells, ANGPTL4 had a more significant effect on oxidative phosphorylation but had a less significant effect on glycolysis.

The main raw materials of tumour cell oxidative phosphorylation are glutamine and fatty acid β oxidation. At present, there are many studies about ANGPTL4 participating in lipid metabolism in the body. Angiopoietin‐like protein (ANGPTL)4 regulates plasma lipids, and ANGPTL4 deficiency increases lipid uptake and respiration in macrophages.^25^ ANGPTL4 presented a negative correlation with BMI, waist circumference, weight and insulin.^26^ ANGPTL4 is involved in the regulation of lipid metabolism in metabolic syndrome, obesity, diabetes and cardiovascular disease.^27^ However, there are few studies on the regulation of lipid metabolism by ANGPTL4 in tumour cells. Using isotope labelling, we investigated the effect of ANGPTL4 on glutamine metabolites and fatty acid oxidation in NSCLC cells. The results showed that the contents of glutamine metabolites (a‐KG (M+5), aspartate (M+4) and malate) decreased significantly after knockdown of ANGPTL4. Glutamine is converted into glutamic acid, which is catalysed to generate α‐ketoglutarate (α‐KG), which participates in the mitochondrial TCA cycle. Our studies have found that ANGPTL4 has no significant effect on glycolysis in NSCLC. The isotope labelling results showed that ANGPTL4 significantly affected the production of acetyl‐CoA from lipid metabolism. This study shows that ANGPTL4 can promote the synthesis of fatty acids in NSCLC, so the effect of fatty acids in tumour cells on ANGPTL4 expression and its mechanism need to be further studied.

To further clarify the effect of ANGPTL4 on tumour cell energy metabolism, an implanted tumour animal model was established. Nude mice were subcutaneously inoculated with A549 cells. When pshRNA‐ANGPTL4 was given to mice, ANGPTL4 expression and the tumour were significantly reduced, and tumour energy metabolism was simultaneously significantly decreased. The contents of glutathione and acetyl‐CoA in the tumours of nude mice were decreased significantly, but there was little effect on lactate. RNA microarray analysis showed that knockdown of ANGPTL4 has certain effects on fatty acid oxidation, glutamine metabolism and the glycolysis pathway. The gene expression of CPT1 and GLS was significantly decreased. To further clarify the pathway by which ANGPTL4 regulates energy metabolism, ANGPTL4‐overexpressing cells were cultured in medium with or without glutamine, and the effects on NSCLC cell proliferation were strikingly decreased in glutamine‐free medium compared to glutamine medium. However, compared with normal cells, there were still significant differences in the glutamine‐free medium. In ANGPTL4‐overexpressing cells, the regulatory effect on glutamine metabolism and fatty acid metabolites disappeared following treatment with both CPT1 and GLS inhibitors, and there was no significant difference in the proliferative activity between ANGPTL4‐overexpressing cells and normal NSCLC cells. Some studies have shown that although omega‐3 polyunsaturated fatty acids (ω‐3 PUFAs) suppress ROS production by enhancing antioxidant stress^28^ and induce autophagy‐mediated cell death in cancer cells, supporting their use as adjuvant therapeutic agents for the treatment of various human cancers,^29^ ω‐3 PUFAs have the highest potency to induce ANGPTL4.^30^ Whether ω‐PUFAs participate in tumour energy metabolism and inhibit tumour cell proliferation through ANGPTL4 remains to be further clarified.

In conclusion, ANGPTL4 is involved in glutamine metabolism and lipid metabolism, which can affect the occurrence and development of many chronic diseases. In this study, we found that ANGPTL4 can affect the energy metabolism of NSCLC cells, and the main mechanism is to influence CPT1 and GLS in NSCLC cells and then regulate glutamine metabolism and fatty acid oxidation rather than glycolysis.

## CONFLICTS OF INTERESTS

The authors declare that there are no conflicts of interest.

## AUTHOR CONTRIBUTIONS

**song xiao:** Conceptualization (lead); Writing‐review & editing (lead). **Nai‐dong Wang:** Data curation (lead); Methodology (equal). **jin‐xiang Yan:** Data curation (equal); Methodology (equal). **long tian:** Formal analysis (equal); Methodology (equal). **xiurong lu:** Data curation (equal). **hong Gao:** Formal analysis (equal); Investigation (equal). **jie‐cheng Yan:** Formal analysis (equal). **fei Zhang:** Methodology (equal).

## ETHICS APPROVAL

Not applicable.

## CONSENT FOR PUBLICATION

Not applicable.

## Supporting information

Supplementary Material

## Data Availability

The data sets during and/or analysed during the current study available from the corresponding author on reasonable request.
