# Supplementary material for: ANGPTL4 regulate glutamine metabolism and fatty acid oxidation in nonsmall cell lung cancer cells
Source: J Cell Mol Med. 2022 Mar 13;26(7):1876–85. doi: 10.1111/jcmm.16879 (PMC8980907; doi:10.1111/jcmm.16879)
Supplement: Supplementary file 1 — Supplementary Material [file JCMM-26-1876-s001.docx]

Supplement 1

Table1S. The results of RNA microarray analysis of ANGPTL4 on fatty acid oxidation, glutamine metabolism and glycolysis pathway gene expression

| Ensembl.ID | Symbol | Regulation | FC (abs) |
| --- | --- | --- | --- |
| ENSG00000105281 | SLC1A5 | Up | 1.657413 |
| ENST00000618632 | SLC1A3 | Up | 1.068127 |
| ENSG00000115419 | GLS | Down | 12.487622 |
| ENSG00000148672 | GLUD1 | Up | 2.100324 |
| ENSG00000133703 | KRAS | Up | 2.114652 |
| ENSG00000110090 | CPT1 | Down | 7.842796 |
| ENSG00000115361 | ACADL | Up | 1.334745 |
| ENSG00000072778 | ACADVL | Up | 1.006651 |
| ENSG00000159399 | HK2 | Up | 3.415211 |
| ENSG00000067225 | PKM | Up | 1.254867 |
| ENSG00000170525 | PFKFB3 | Down | 1.324162 |
| ENSG00000005882 | PDK2 | Down | 1.065873 |
| ENSG00000228612 | [HK2P1](http://asia.ensembl.org/homo_sapiens/Gene/Summary?g=ENSG00000228612&db=core) | Down | 1.167348 |
| ENSG00000156510 | HKDC1 | Up | 2.847612 |


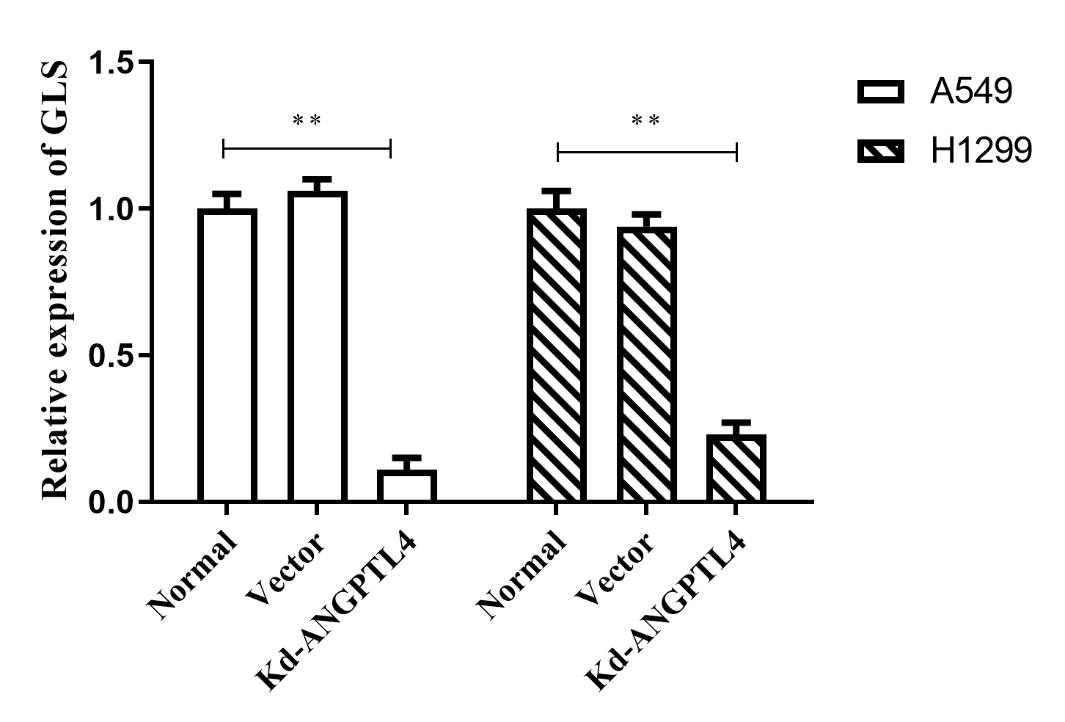


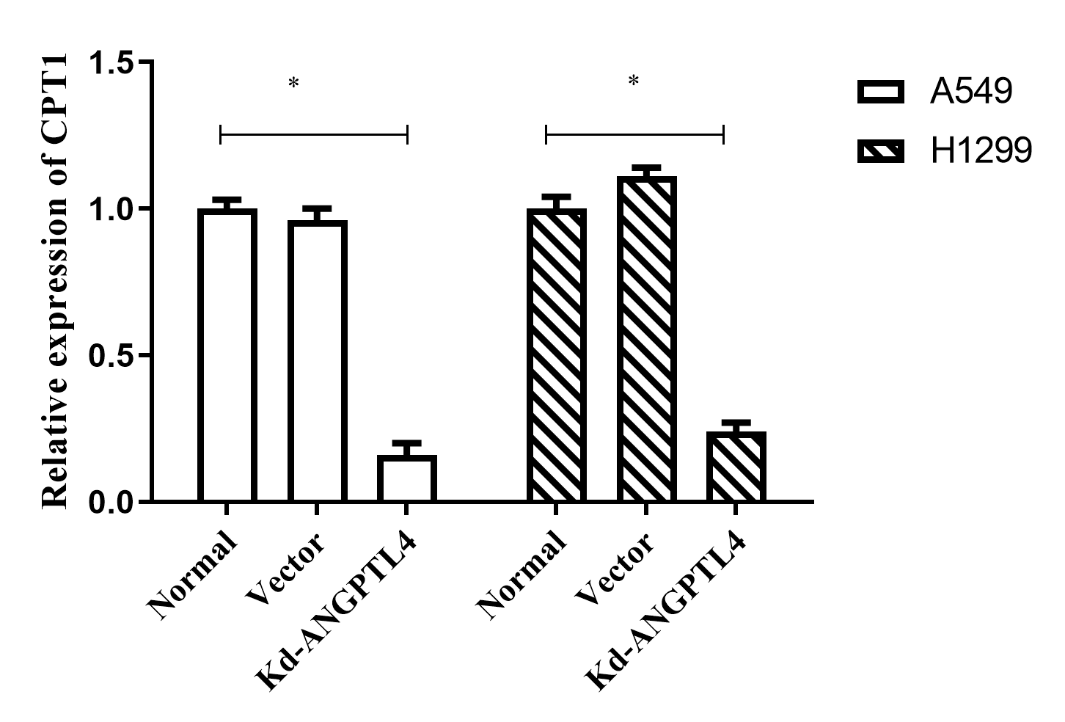


Fig1S. The gene expression of CPT1 and GLS in A549 and H1299 cell *P<0.05.
